# Considering Biomarkers of Neurodegeneration in Alzheimer’s Disease: The Potential of Circulating Cell-Free DNA in Precision Neurology

**DOI:** 10.3390/jpm14111104

**Published:** 2024-11-13

**Authors:** Chad A. Pollard, Erin R. Saito, Jeffrey M. Burns, Jonathon T. Hill, Timothy G. Jenkins

**Affiliations:** 1 Department of Cell Biology and Physiology, Brigham Young University, Provo, UT 84602, USA; 2 Resonant, Heber, UT 84032, USA; 3 University of Kansas Alzheimer’s Disease Research Center, Fairway, KS 66205, USA

**Keywords:** neurodegenerative disease, Alzheimer’s disease, cell-free DNA, DNA methylation, precision neurology

## Abstract

Neurodegenerative diseases, such as Alzheimer’s disease (AD), are a growing public health crisis, exacerbated by an aging global population and the lack of effective early disease-modifying therapies. Early detection of neurodegenerative disorders is critical to delaying symptom onset and mitigating disease progression, but current diagnostic tools often rely on detecting pathology once clinical symptoms have emerged and significant neuronal damage has already occurred. While disease-specific biomarkers, such as amyloid-beta and tau in AD, offer precise insights, they are too limited in scope for broader neurodegeneration screening for these conditions. Conversely, general biomarkers like neurofilament light chain (NfL) provide valuable staging information but lack targeted insights. Circulating cell-free DNA (cfDNA), released during cell death, is emerging as a promising biomarker for early detection. Derived from dying cells, cfDNA can capture both general neurodegenerative signals and disease-specific insights, offering multi-layered genomic and epigenomic information. Though its clinical potential remains under investigation, advances in cfDNA detection sensitivity, standardized protocols, and reference ranges could establish cfDNA as a valuable tool for early screening. cfDNA methylation signatures, in particular, show great promise for identifying tissue-of-origin and disease-specific changes, offering a minimally invasive biomarker that could transform precision neurology. However, further research is required to address technological challenges and validate cfDNA’s utility in clinical settings. Here, we review recent work assessing cfDNA as a potential early biomarker in AD. With continued advances, cfDNA could play a pivotal role in shifting care from reactive to proactive, improving diagnostic timelines and patient outcomes.

## 1. Introduction

### 1.1. The Critical Need for Early Detection

Neurological disorders are now recognized as the leading cause of global health burden [1]. Among these, neurodegenerative diseases pose a rapidly escalating crisis, especially as the global population ages, with no effective therapies available to halt their progression [2]. Alzheimer’s disease (AD), the most common neurodegenerative disease and the seventh leading cause of death in the U.S., affects approximately 6.7 million Americans alone, a number expected to nearly triple in the next 30 years [3]. Other neurodegenerative conditions, all with life-altering impacts, affect millions more.

As recently as 1990, a definitive diagnosis of AD and many other neurodegenerative conditions could only be made postmortem following brain tissue biopsies [4,5]. Although postmortem evaluation remains the gold standard, diagnostic criteria have evolved with the advent of biomarker and imaging technologies that enable the earlier identification of brain pathology in living patients [6]. However, these tools are typically applied after clinical symptoms have emerged. By the time hallmark symptoms—such as memory loss in AD—become apparent, it is well established that substantial and often irreversible neuronal damage has already occurred [7,8]. Many experts believe the high failure rate of clinical trials for neurodegenerative diseases stems from targeting the pathologies too late in the disease course when too many neurons have been lost to meaningfully slow the disease [9].

Consistent with these hypotheses, delaying symptom onset by as little as five years has been projected to reduce the prevalence of AD by up to 50% [10]. Therefore, delaying disease onset via early detection and intervention is now recognized as a key strategy for mitigating the overall burden of neurodegenerative disorders [11]. Research shows that the pathological processes driving AD begin years to decades before symptoms surface [12]. Recent insights into the early onset of AD pathology and the potential benefits of presymptomatic treatment have driven revisions in several diagnostic criteria to reflect AD-specific neuropathology and utilize general biomarkers of neurodegeneration for disease staging [13,14,15]. These changes are enabling the identification of less invasive biomarkers with the potential to detect neurodegenerative mechanisms in earlier stages.

### 1.2. Comparing cfDNA with Other Biomarkers of AD and Neurodegeneration

Compared to genetic risk factors and other biomarkers of AD and neurodegeneration, cell-free DNA (cfDNA) stands out as a promising candidate for early detection. While genetic risk factors have been identified for many neurodegenerative conditions, they do not capture active disease processes and are thus insufficient for detecting or monitoring disease progression. In sporadic AD, the apolipoprotein E ε4 (APOE4) allele is a well-known risk factor, significantly increasing the likelihood of developing the disease [16]. However, although 40–65% of AD patients carry at least one APOE4 allele, its presence is neither necessary nor sufficient for diagnosis—many carriers remain unaffected, while some non-carriers still develop AD.

In the shift toward a biological definition of Alzheimer’s disease (AD) [13], several AD-specific neuropathology biomarkers, such as amyloid and tau, have been proposed as core diagnostic criteria and potential targets for early detection in presymptomatic individuals. However, these protein biomarkers show limited sensitivity for detecting early, mild AD compared to neuropathologic examination [17]. This limitation is significant, as the overlap of AD pathology with other neurodegenerative and age-related conditions can complicate early diagnosis, making it difficult to distinguish early AD from other cognitive impairments. Because thoroughly validated biomarkers are not available for all related neurodegenerative diseases, ruling out other diagnoses through elimination remains challenging.

Given the limited sensitivity and potential overlap with other mechanisms and diagnoses, core AD biomarkers—amyloid and tau—are primarily recommended for evaluating symptomatic individuals rather than cognitively unimpaired populations [13]. This approach remains controversial, as early detection and intervention are widely viewed as crucial strategies for reducing the burden of neurodegenerative diseases [11]. Achieving accurate, presymptomatic diagnosis requires sensitive and specific biomarkers that can detect AD processes before significant neuronal damage occurs, without risking false positives and unnecessary intervention in individuals who may not develop symptoms.

While amyloid and tau biomarkers are valuable for AD diagnosis, they are limited in scope as early screening and disease monitoring targets. Amyloid deposits, for instance, may occur decades before symptoms but do not correlate well with cognitive performance; thus, amyloid measures alone are unreliable for predicting cognitive decline [18,19]. For example, 10–30% of cognitively normal elderly individuals display amyloid deposition in the brain [20]. Tau, which is more closely linked to clinical outcomes, often appears too late to capture early neurodegeneration [21]. Additionally, some data suggest these proteins may even be byproducts of neurodegeneration rather than primary indicators of disease [22,23], underscoring the need for more precise biomarkers.

General neurodegeneration biomarkers like glial fibrillary acidic protein (GFAP), a marker of astrocytic injury [24], and neurofilament light chain (NfL), a marker of axonal degeneration [25], also have limitations. Though useful for monitoring general neurodegeneration, they lack the specificity required for actionable, disease-specific insights. For example, NfL is sensitive to various forms of neurodegeneration but cannot distinguish between specific disease processes or cell types affected, limiting its utility for early, targeted interventions. GFAP, while valuable in detecting astrocytic damage, similarly lacks the specificity needed to pinpoint neuronal damage or predict disease progression at the cellular level. NfL and GFAP primarily provide single-measure insights into inflammation and axonal integrity, limiting their utility for detailed disease characterization or early-stage detection across specific neurodegenerative pathways or specific diseases.

In contrast, circulating cell-free DNA (cfDNA) offers several unique advantages. Derived from dying neurons, cfDNA may detect both broad neurodegenerative signals and provide disease-specific insights across neurodegenerative diseases [26,27,28]. Released from dying neurons, cfDNA’s potential for identifying tissue-of-origin through methylation signatures allows for greater cell-type specificity, providing the potential to distinguish between different neuronal cell types and disease states. Unlike NfL and GFAP, cfDNA captures multiple layers of genomic and epigenomic information, which can reflect both the progression and molecular characteristics of specific neurodegenerative processes. By combining cfDNA data with additional diagnostic indicators, such as protein aggregates like amyloid in AD, clinicians could gain a more comprehensive view of disease pathology, which may enhance diagnostic accuracy and support more targeted, personalized treatment approaches for early disease detection.

Though still under investigation, the clinical utility of cfDNA is supported by ongoing advances in detection sensitivity, reference range development, and protocol standardization, all of which could strengthen its role in early neurodegenerative disease screening to improve diagnostic timelines and patient outcomes. While cfDNA in cerebrospinal fluid (CSF) is also being explored [29,30], this review focuses on blood plasma cfDNA due to its accessibility, minimally invasive nature, and potential for broader clinical integration [31]. We emphasize cfDNA methylation signatures as particularly promising for clinical applications due to their stability in circulation across varied conditions [32], ability to identify cells of origin and disease-specific alterations [33,34], and relevance in multiple therapeutic areas, including oncology [34,35,36,37,38].

### 1.3. cfDNA Biology and Its Emerging Role in Neurodegenerative Disease

cfDNA consists of small DNA fragments released into circulation following cell death [39]. These fragments vary in size, with a peak length of 167 base pairs, corresponding to the approximate length of DNA wrapped around a single nucleosome [40]. cfDNA can be released passively through apoptosis (programmed cell death), necrosis (uncontrolled cell death), or actively via the secretion of extracellular vesicles [40,41]. These fragments carry a wealth of genetic and epigenetic information, including somatic and germline genetic variants, epigenetic modifications, and informative nucleosome spacing patterns, which are increasingly being leveraged in research and clinical settings to understand biological mechanisms. 

cfDNA’s multi-omic nature has expedited its application in diverse clinical contexts, including prenatal testing [42,43], cancer diagnostics [44,45,46,47,48,49,50,51,52], transplant rejection monitoring [53,54], and cardiovascular disease management [55]. As a dynamic biomarker, cfDNA offers real-time insights into cellular turnover due to its short half-life, which ranges from 16 min to 2.5 h [44]. In healthy individuals, cfDNA levels remain relatively low and comprise DNA primarily derived from hematopoietic cells [33]. However, during significant cellular death, as seen in cancer or neurodegenerative disorders, cfDNA levels increase markedly, reflecting tissue damage.

In the context of neurodegenerative diseases such as AD, neuron-derived cfDNA fragments circulating in the bloodstream may provide an early indicator of neuronal death or injury [26]. Analyzing methylation patterns, nucleosome spacing [56,57], and other genetic modifications in blood cfDNA can allow researchers to trace these fragments back to neuronal tissue, potentially detecting neurodegenerative processes long before clinical symptoms appear. This positions circulating cfDNA as a versatile, minimally invasive biomarker with broad potential for early disease detection across the neurodegenerative spectrum.

While other aspects of cfDNA biology, like fragmentomics [58] and nucleosome spacing, and other circulating nucleic acids, such as miRNAs and mtDNA, have shown promise as relatively stable and informative biomarkers [59,60,61], they have been recently reviewed elsewhere [62,63]. Additionally, limitations in tissue specificity and susceptibility to external factors, such as inflammation or stress, may hinder their utility for early detection and disease monitoring in AD. miRNAs, for instance, play roles in various cellular processes, making it difficult to pinpoint specific tissue sources or disease states [64]. Similarly, mtDNA reflects general cellular damage but lacks the detailed specificity required to identify neuron-specific pathways [65,66].

In contrast, cfDNA methylation provides more granular information due to its high specificity for tissue of origin [33] and its ability to reveal disease-specific epigenetic changes [34]. This dual functionality makes it a particularly promising candidate for early detection and driving targeted therapeutic interventions in AD.

## 2. Learning from Oncology: cfDNA Biomarkers as Early Detection Tools

Advances in neuro-oncology provide a promising foundation for developing cfDNA-based liquid biopsies to advance precision medicine in neurology. Over the past two decades, circulating tumor DNA (ctDNA), a specific subtype of cfDNA released by tumor cells, has driven significant advances in cancer diagnostics [67]. Because cancer is an inherently a complex, multifactorial disease—characterized by mutations, structural variants, and epigenetic modifications that disrupt cell regulation and drive rampant cell growth and proliferation—blood-based ctDNA liquid biopsies have emerged as minimally invasive tools for capturing and monitoring these molecular changes in real time [68]. Although challenges related to standardization and technical validation remain to be addressed before cfDNA assays can be widely implemented, clinical trials demonstrate promising results and confirm cfDNA as a potential tool for real-time monitoring of acquired resistance, disease progression, and treatment selection, extending the overall survival of cancer patients [47].

With growing evidence suggesting impactful roles for cfDNA-based biopsies for peripheral tumors, ctDNA is increasingly looked to for detecting and managing brain tumors, which are inaccessible via traditional tissue biopsies. Initially, it was believed that brain tumors released little to no ctDNA, making detection in blood plasma challenging to impossible. However, recent studies have demonstrated that ctDNA from brain tumors, including gliomas, can be detected in peripheral blood [69]. Although ctDNA was detected in fewer than 10% of glioma patients in this early study, it provided proof of concept that with improved detection technologies, ctDNA could revolutionize diagnostics and monitoring for brain cancers. More recent work has shown that, even in healthy individuals, brain-derived cfDNA makes up approximately 2% of circulating cfDNA [33]. Subsequent research utilizing next-generation sequencing (NGS) has revealed somatic ctDNA alterations in over 50% of primary brain tumors (meningioma 59%, glioblastoma 55%) [70].

Advances in ctDNA technologies have also facilitated the identification of epigenetic modifications, such as DNA methylation, enabling researchers to trace ctDNA to its tissue or cell of origin [71]. In a study involving 220 primary brain tumor samples, plasma ctDNA methylation profiles successfully differentiated various brain tumor types that are often difficult to distinguish using imaging techniques, including gliomas, meningiomas, and glial–neuronal tumors [72]. This capacity to differentiate between complex tumor types underscores the growing potential of cfDNA methylation profiles as robust biomarkers in neurology.

Although cell-of-origin technologies have yet to obtain regulatory approval, these developments in ctDNA-based diagnostics highlight the potential for genomically driven, personalized treatment decisions in oncology, which are actively being investigated [73]. Furthermore, they pave the way for similar breakthroughs in precision medicine for AD and other neurodegenerative diseases, where early detection and targeted interventions are critically needed. The principles of ctDNA diagnostics from oncology can be adapted to AD. While cancers and neurodegenerative diseases differ in their underlying causes, both involve cellular dysfunction and degeneration—the uncontrolled growth, proliferation, and death of cancer cells or the progressive loss of neurons.

## 3. Neurodegeneration: cfDNA as a Potential Biomarker for Alzheimer’s Disease

Although the vast majority of AD cases are not primarily driven by genetic mutations, cfDNA-based liquid biopsies hold great promise for transforming patient care. While cfDNA research in neurodegenerative disorders is still in the early stages, lagging behind its application in neuro-oncology, emerging studies have demonstrated that neuron-derived cfDNA can be detected in the blood and analyzed as a potential early biomarker of AD [26]. These early studies can be found in Table 1 and are laying the groundwork for future cfDNA advances in early diagnosis, personalized treatment, and disease monitoring.

For instance, elevated cfDNA levels correlate with a higher risk of cognitive decline. A large longitudinal study of 631 cognitively healthy individuals (average age of 80) found that those with higher levels of total cfDNA in the blood experienced steeper cognitive decline and worsening frailty over an eight-year period [74]. Although this study highlights the potential relevance of cfDNA liquid biopsies to neurodegenerative disorders, it quantified total cfDNA by targeting broadly conserved regions of nuclear DNA, leaving their neurological significance and specific tissue origin unknown. More targeted research is needed to link cfDNA directly to the neurodegenerative processes specific to AD.

In a more targeted study, our lab demonstrated that neuron-derived cfDNA can distinguish individuals with AD and mild cognitive impairment (MCI) from cognitively healthy controls [75]. Using a targeted bisulfite sequencing approach that assessed methylation at a single neuron-specific cfDNA site, this small-scale study (*n* = 50) revealed that patients with AD and individuals with MCI who progressed to AD within five years exhibited higher levels of cortical-neuron-derived cfDNA in the blood plasma. Although promising, these findings highlight the need for larger, longitudinal cell-of-origin studies and more sensitive technologies to validate the clinical utility of neuron-derived cfDNA as a reliable marker for early detection and disease monitoring in AD.

DNA methylation and other epigenetic modifications, which play key roles in regulating gene expression, have been extensively studied as potential drivers of neurodegenerative disease [76]. Beyond their utility in cell-of-origin analyses, disease-specific methylation signatures are also emerging as promising diagnostic tools. In addition to tracing cfDNA’s tissue of origin, analyzing methylation patterns can also reveal epigenetic changes associated with specific disease processes. For example, Pai et al. identified elevated total cfDNA levels and a unique methylation signature in *LHX2*, a gene critical for neuronal development, in patients with AD [77]. Differential *LHX2* methylation displayed early-stage biomarker potential, a finding supported by a more recent study that used targeted bisulfite sequencing and also found differential methylation of a CpG site within *LHX2* in AD blood plasma [78]. These findings suggest that cfDNA methylation can capture both the origin of the neuronal damage and disease-specific changes. However, more extensive studies are needed to validate the robustness of *LHX2* and other similar methylation markers for clinical application.

A genome-wide DNA methylation study of circulating cfDNA further supports the potential of cfDNA methylation analysis for AD diagnostics. This study identified 3684 differentially methylated cfDNA sites in AD patients compared to controls [79]. Pathway enrichment analysis revealed that these methylation changes were disproportionately associated with genes involved in key biological pathways linked to AD pathology, including calcium signaling, glutamatergic synapse, and hedgehog signaling. By applying six artificial intelligence (AI) platforms, the researchers identified five best-performing methylation sites that achieved high diagnostic accuracy for AD prediction with an AUC of 0.99—underscoring the potential of cfDNA methylation as a powerful tool for early AD detection.

Building on these findings, a study by Chen et al. further explored the potential of methylation modifications as diagnostic biomarkers for AD, focusing on hydroxymethylation [80]. The study identified 19 genes with differential hydroxymethylation in patients with late-onset AD compared to controls. Pathway enrichment analysis revealed these differentially methylated genes were primarily involved in pathways linked to AD pathogenesis, such as tight junctions, MAPK signaling, dopaminergic synapses, neurotrophic signaling, and NF-kappa B signaling. Notably, five of these genes—*RABEP1*, *CPNE4*, *DNAJC15*, *REEP3*, and *ROR1*—were negatively correlated with cognitive performance, as measured by the Mini-Mental State Examination (MMSE) and Montreal Cognitive Assessment (MoCA). Their hydroxymethylation levels were significantly elevated in the blood cfDNA of AD patients. An ROC analysis demonstrated a high diagnostic accuracy, with an AUC of 0.921. These findings suggest that abnormal hydroxymethylation modifications in these genes may also serve as valuable biomarkers for distinguishing AD patients from cognitively normal individuals.

In another study of cognitively normal individuals and patients with MCI, researchers identified differential methylation patterns across up to 19 DNA regions associated with cognitive decline and conversion to MCI [81]. However, no single CpG site demonstrated sufficient clinical utility as a standalone biomarker, reinforcing the idea that more complex methylation biosignatures, rather than individual sites, may be required for accurate prediction and diagnosis. Expanding this research to larger populations and incorporating multi-omic approaches could help uncover more comprehensive cfDNA-based biosignatures.

While AD has been a primary focus of cfDNA research in neurodegenerative disorders, similar approaches are also being explored in other conditions. For example, a smaller-scale study in Parkinson’s disease (PD) patients also revealed elevated cfDNA levels in blood serum compared to healthy controls, suggesting potential applications for cfDNA in monitoring PD as well [82].

cfDNA cell-of-origin methylation analysis has also shown promise in other neurological conditions. In a study similar to that of Pollard et al. [75], Lehmann-Werman et al. demonstrated that cfDNA methylation patterns can also act as tissue-specific biomarkers in multiple sclerosis (MS) and traumatic brain injury (TBI) [83]. Using targeted bisulfite sequencing, the study found elevated levels of oligodendrocyte-derived cfDNA in the blood of MS patients and brain-derived cfDNA in TBI patients. These findings suggest that cfDNA methylation could be a powerful tool for identifying cell-specific damage in AD and other neurological conditions, underscoring its broad potential in neurodegenerative disease detection and monitoring. Additional cfDNA-based studies in epilepsy [84], multiple sclerosis [85], and other disorders [86] are underway, further validating the presence of neuron-derived cfDNA in peripheral circulation across a range of conditions. These efforts collectively strengthen the case for developing cfDNA-based screening tools.

While these early studies suggest great promise, much work remains to refine cfDNA-based biomarkers for AD and other neurodegenerative diseases. The development of screening tools tailored to detecting neuron-derived cfDNA, combined with advances in methylation analysis, could significantly enhance early detection and personalized treatment in neurology. Furthermore, ongoing research in cfDNA methylation profiling for precision neuro-oncology [69,72] may offer valuable insights that can be adapted for other neurological conditions. As this emerging field advances, larger cohort studies, better characterization of cell-specific methylation patterns, and the integration of multi-omic approaches will be critical to unlocking the full potential of cfDNA in precision neurology for neurodegenerative diseases.

**Table 1:** Summary of key studies on cfDNA as a biomarker for Alzheimer’s disease. This table presents selected studies investigating cfDNA-based liquid biopsies for Alzheimer’s disease. Methods used, key findings showcasing the potential of cfDNA methylation and hydroxymethylation as potential targets for early screening, and references are included.

Condition	Methods	Key Findings	Reference
Alzheimer’s Disease	Targeted bisulfite sequencing	Higher levels of cortical-neuron-derived cfDNA detected in AD and MCI	Pollard et al., 2023 [75]
	Targeted bisulfite sequencing for *LHX2* gene	Differential methylation of LHX2 in AD patients, showing early-stage biomarker potential	Pai et al., 2019 [77]
	AI-assisted methylation array analysis	3684 differentially methylated sites with high AD prediction accuracy (AUC = 0.99)	Bahado-Singh et al., 2022 [79]
	Differential 5 hmC profiling via chemical labeling	Elevated 5 hmC levels in AD-related genes correlated with cognitive decline	Chen et al., 2022 [80]
Cognitive Decline	Total blood cfDNA quantification via digital PCR	Elevated cfDNA linked to cognitive decline and frailty over eight years	Nidadavolu et al., 2022 [74]

## 4. Technological Advances: The Challenge of cfDNA in Clinical Diagnostics

### 4.1. Limitations of Current Technologies

While these early studies underscore the potential of cfDNA as a biomarker, challenges remain regarding detection sensitivity and the need for further technological advances. One of the significant challenges with cfDNA-based assays is the fact that cfDNA naturally occurs in low concentrations in circulation. Brain-derived cfDNA is even more sparse [33,83]. This low abundance makes detecting and analyzing cfDNA as an informative biomarker particularly difficult, especially early in disease progression when neurodegeneration rates are low. Thus, a key obstacle to applying and integrating cfDNA-based tests in clinical practice is the need for more advanced technologies with greater sensitivity, accuracy, and precision. Numerous recent reviews have highlighted these technological gaps, emphasizing the improvements required before blood-based cfDNA assays can be meaningfully integrated into clinical diagnostics for neurological disorders [27,44,87,88].

Since its introduction in the early 1990s, bisulfite sequencing has become the de facto standard for methylation analysis [89]. The process involves three main steps: (1) treatment of DNA with sodium bisulfite to differentiate methylated from unmethylated cytosines by converting unmethylated cytosines to uracils, (2) PCR amplification to generate sufficient quantities of DNA, and (3) sequencing followed by bioinformatic analysis to determine the methylation status of cytosines.

In addition to bisulfite sequencing, methylation arrays—such as the Infinium MethylationEPIC array (Illumina, San Diego, CA, USA)—are widely used for methylation analysis due to their standardized protocols and user-friendly platforms [90,91]. These arrays offer a high-throughput, cost-effective means of analyzing hundreds of thousands of CpG sites across the genome. Unlike sequencing-based methods, methylation arrays rely on hybridization-based detection following bisulfite treatment, eliminating the need for PCR amplification or next-generation sequencing (NGS).

Despite the widespread adoption of both bisulfite sequencing and methylation arrays, bisulfite treatment is well known to damage DNA [92], with some reports estimating losses of up to 90% of the DNA template [93]. More recent assessments have demonstrated a post-bisulfite DNA recovery rate of 18–50% [94]. Additionally, PCR amplification used in bisulfite sequencing is known to introduce biases that can distort the quantification of methylation levels, potentially leading to the over- or underestimation of global methylation [95]. While methylation arrays mitigate some of the challenges associated with whole-genome bisulfite sequencing, they are limited to predefined regions of interest and thus may miss informative loci in less-studied genomic regions.

Reviews assessing cfDNA methylation as a potential biomarker for brain disorders outline several current approaches, including whole-genome bisulfite sequencing, reduced-representation bisulfite sequencing, MeDIP-seq, methylated CpG tandem amplification and sequencing, targeted next-generation bisulfite sequencing, and 5 hmC profiling. Despite these advances, these reviews conclude that there are still no clinical applications of cfDNA methylation biomarkers for brain disorders due to limitations in sensitivity [27,44,87,88]. For example, a recent pilot twin study investigated plasma cfDNA methylation as a potential early biomarker for episodic memory impairment with whole-genome bisulfite sequencing [96]. The study included 559 same-sex twin pairs and found no significant markers associated with episodic memory performance. The authors concluded that with the low genomic coverage of cfDNA and inter-individual variability, even if the sample size were increased, bisulfite sequencing is suboptimal for detecting informative biomarkers for early detection of cognitive decline in AD or any other condition. Thus, technologies that eliminate bisulfite treatment, such as Oxford Nanopore Technologies’ nanopore sequencing and PacBio’s single-molecule real-time sequencing (SMRT), have been proposed as promising alternatives.

### 4.2. Emerging (Native-Read) Technologies for cfDNA Profiling

Native, long-read sequencing platforms like Oxford Nanopore Technologies (ONT) and Pacific Biosciences (PacBio) are emerging as powerful tools in genomic and epigenomic profiling with several advantages over traditional NGS [97]. Unlike traditional next-generation sequencing (NGS) methods, these long-read sequencing technologies can directly sequence DNA in its native, unaltered form, preserving epigenetic signatures and enabling their direct detection. For methylation detection, this eliminates the need for bisulfite conversion or PCR, which are known to cause extensive DNA damage and introduce biases [93]. Initially designed for longer DNA reads, ONT and PacBio have adapted their platforms to efficiently sequence shorter DNA fragments like cfDNA with no additional library preparation [98].

When first introduced, long-read sequencing technologies were plagued by high error rates, limiting their utility for research and clinical applications. However, recent advances—particularly in the last 3 to 5 years—have significantly improved accuracy [97]. Technologies like ONT’s updated pore chemistry and PacBio’s HiFi sequencing now offer error rates comparable to traditional short-read platforms like Illumina [99]. This leap in accuracy and the ability to directly detect methylation position native-read technologies as a strong alternative for comprehensive cfDNA methylation analysis.

In parallel, advances in bioinformatics have greatly enhanced the processing of long-read data, addressing challenges related to base calling, methylation detection, and genome assembly [100]. These improvements, combined with the increased accuracy of long-read platforms, are accelerating their transition from research tools to viable clinical solutions [101]. As these technologies continue to evolve, they hold the potential to revolutionize cfDNA profiling and unlock new possibilities for precision medicine in neurodegenerative diseases.

### 4.3. Bridging the Gap: From Research to Clinical Application

Despite its promise, translating cfDNA technologies from research into clinical applications, particularly for neurological disorders, faces several technical and logistical hurdles. One of the major challenges is the sensitivity and accuracy required to detect cfDNA, which is typically found in low concentrations during the early stages of neurodegeneration. Low levels of cfDNA are often difficult to distinguish from background noise, making early detection challenging [50]. Future advances in sequencing accuracy and bioinformatics tools will be essential for distinguishing disease-relevant cfDNA from background signals. While significant improvements have been made in sequencing technologies, such as those developed by ONT and PacBio, further advances are essential to consistently detect subtle epigenetic and genetic alterations with high precision.

Additionally, the clinical implementation of cfDNA-based diagnostics requires cost-effective, high-throughput methods that can be scaled for widespread use in healthcare settings. The pre-analytical variability in cfDNA studies also presents a significant barrier to clinical translation. Variability in cfDNA levels due to factors like sample storage and processing can obscure meaningful differences between healthy and diseased subjects [102]. Standardization of pre-analytical protocols—such as the choice of storage tubes, timing of blood draws, and handling conditions—will ensure that cfDNA tests yield consistent, reliable results across clinical settings.

In addition to technical standardization, the field needs to better understand cfDNA dynamics throughout disease progression. One of the most pressing needs is the establishment of longitudinal studies that track cfDNA biomarkers over time. Current research relies heavily on small-scale, cross-sectional studies, which offer valuable snapshots but do not capture how cfDNA dynamics evolve as neurodegeneration progresses. Large-scale longitudinal studies are necessary to validate the clinical utility of cfDNA-based tests. Such studies will help establish reference ranges for cfDNA levels in different patient populations and identify patterns that could inform presymptomatic intervention strategies.

While much work remains, cfDNA-based diagnostics hold the potential to be transformative in clinical practice. The ability to detect neuron-derived cfDNA and its epigenetic markers in real time offers the possibility of earlier disease detection, more personalized treatment plans, and more precise disease monitoring. Continued technological advances and comprehensive longitudinal studies will be vital to overcoming the current challenges and unlocking the full potential of cfDNA biomarkers for precision neurology.

## 5. Conclusions

cfDNA has emerged as a promising biomarker for the early detection of neurodegeneration in AD and other neurodegenerative diseases. It holds the potential to bridge critical diagnostic gaps by capturing genetic and epigenetic signatures from dying neurons, potentially facilitating earlier intervention. Current diagnostic tools for AD often detect disease after substantial neuronal damage has occurred. cfDNA’s ability to provide real-time insights into disease progression through methylation analysis and other modifications could fundamentally reshape how we identify and manage AD and other conditions.

Despite this promising outlook, several challenges must be addressed before cfDNA biomarkers can be routinely used in clinical practice for early AD detection. Enhancing detection sensitivity and accuracy is essential to identify early-stage AD from the small cfDNA concentrations characteristic of neurodegenerative diseases [50]. Standardizing pre-analytical protocols to reduce variability across clinical settings and achieving cost-effectiveness and scalability will be crucial for the widespread adoption of cfDNA-based tests [102]. Additionally, comprehensive longitudinal studies are needed to validate cfDNA biomarkers, establish reference ranges, and improve our understanding of cfDNA dynamics throughout the progression of AD.

While still in development, the potential for cfDNA-based technologies in AD diagnostics is substantial. Presymptomatic screening tools are urgently needed to shift care from a reactive to a proactive model, where disease processes can be identified and addressed before symptoms emerge. With successful validation, cfDNA could play a pivotal role in detecting neurodegeneration early and guiding personalized treatment strategies. As more effective early-stage therapies are developed, cfDNA technologies may help delay the onset of AD, improve patient outcomes, and reduce the global disease burden. Continued research and development are essential to unlock cfDNA’s full potential in precision neurology, addressing one of modern healthcare’s most pressing challenges.

## Data Availability

No new data were created or analyzed in this study. Data sharing is not applicable to this article.
